# Birds and Viruses at a Crossroad - Surveillance of Influenza A Virus in Portuguese Waterfowl

**DOI:** 10.1371/journal.pone.0049002

**Published:** 2012-11-07

**Authors:** Conny Tolf, Daniel Bengtsson, David Rodrigues, Neus Latorre-Margalef, Michelle Wille, Maria Ester Figueiredo, Monika Jankowska-Hjortaas, Anna Germundsson, Pierre-Yves Duby, Camille Lebarbenchon, Michel Gauthier-Clerc, Björn Olsen, Jonas Waldenström

**Affiliations:** 1 Centre for Ecology and Evolution in Microbial Model Systems (EEMiS), Linnæus University, Kalmar, Sweden; 2 Departamento de Recursos Florestais, Escola Superior Agrária, Instituto Politécnico de Coimbra, Coimbra, Portugal; 3 Department of Animal Health, Norwegian Veterinary Institute, Oslo, Norway; 4 Universite de La Reunion, BP7151, Saint-Denis, La Reunion; 5 Centre de Recherche de la Tour du Valat, Le Sambuc, Arles, France; 6 Section of Infectious Diseases, Department of Medical Sciences, Uppsala University, Uppsala, Sweden; Auburn University, United States of America

## Abstract

During recent years, extensive amounts of data have become available regarding influenza A virus (IAV) in wild birds in northern Europe, while information from southern Europe is more limited. Here, we present an IAV surveillance study conducted in western Portugal 2008–2009, analyzing 1653 samples from six different species of waterfowl, with the majority of samples taken from Mallards (*Anas platyrhynchos*). Overall 4.4% of sampled birds were infected. The sampling results revealed a significant temporal variation in the IAV prevalence, including a pronounced peak among predominantly young birds in June, indicating that IAV circulate within breeding populations in the wetlands of western Portugal. The H10N7 and H9N2 subtypes were predominant among isolated viruses. Phylogenetic analyses of the hemagglutinin and neuraminidase sequences of H10N7, H9N2 and H11N3 virus showed that sequences from Portugal were closely related to viral sequences from Central Europe as well as to IAVs isolated in the southern parts of Africa, reflecting Portugal’s position on the European-African bird migratory flyway. This study highlights the importance of Portugal as a migratory crossroad for IAV, connecting breeding stationary waterfowl with birds migrating between continents which enable transmission and spread of IAV.

## Introduction

Wild birds, and in particular dabbling ducks of the genus *Anas*, represent the main reservoir for avian influenza A virus (IAV) [1], [2]. A large diversity of IAV has been described based on genetic differences of two surface proteins, the hemagglutinin (HA, 16 variants) and neuraminidase (NA, 9 variants) [1], [3]. In wild ducks, the majority of IAV strains cause mild infections [4], and are therefore referred to as low pathogenic avian influenza (LPAI). Influenza virus also infects poultry, resulting occasionally in a markedly increased virulence, leading to highly contagious, often fatal disease referred to as highly pathogenic avian influenza (HPAI). HPAI infections cause substantial economic damage for the poultry industry. Regional outbreaks of HPAI in poultry have occurred repeatedly during the last decades, but the emergence of the Asian lineage of H5N1 HPAI in 1999 sparked a near global epizootic in poultry, with several outbreaks in Eurasia and Africa [5], [6], [7], [8], [9], [10], as well as many human fatalities [11], [12], [13]. The H5N1 is unique in that it has not only affected poultry but also caused serious disease in wild birds [5], [7], [14], [15], and it has been argued that wild birds may be responsible for its westward spread in Europe in 2006 [14], [16], [17], [18]. Along with HPAI variants of the H5 and H7 subtypes, the H9N2 virus has caused substantial problems for the poultry industry worldwide, as well as showing a potential to infect pigs and humans [19], [20].

Long-term surveillance studies conducted in Europe [3], [21], [22], [23], [24] and North America [25], [26] have provided key information about IAV epidemiology in wild ducks. IAV prevalence in waterfowl follows a repeatable seasonal pattern, with high prevalence in autumn, during migration, followed by a marked drop in winter, and only a few infected birds in the spring [3], [22], [27]. Studies performed in the Mediterranean region have shown that these wintering grounds for ducks have an impact on the epidemiology of avian influenza [28], [29], [30], [31], and that IAV seems to be present there all year around [32]. In addition, genetic analyses have revealed evidence of intercontinental transmission of viral gene segments between North America and the Mediterranean region [33]. The Western Mediterranean area is a major winter ground for waterfowl, harboring millions of birds each winter. Migratory populations of Eurasian Teals (*Anas crecca*), Eurasian Wigeons (*Anas Penelope*), Mallards (*Anas platyrynchos*) and other anatids winter at these sites and mix with resident bird populations, thereby connecting birds from vast geographic regions, extending from Iceland to Siberia [3], [22], [34], [35]. The connections between the region's wetlands through short-range migration in addition to their role as a stopover site for birds migrating between Europe and Africa makes them particularly interesting for IAV surveillance.

**Figure 1:**
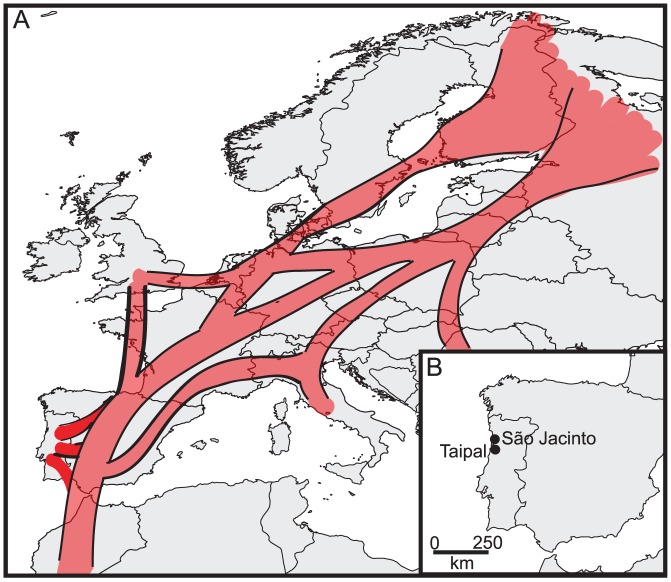
(A) General migration routes of ducks across Europe [**58**], including birds migrating from north eastern summer areas to southern wintering grounds in Southern Europe, and Africa. Different bird species and populations use different migratory routes, stop over locations, and winter locations. (B) Sampling locations in Portugal utilized in this study.

The aim of this study was to investigate prevalence, subtype diversity, and phylogenetic relatedness of IAVs circulating in wild ducks in Western Portugal in context with IAV sequence information collected in public databases. Specifically, we present data from a surveillance project in Portugal and discuss the results in the light of recent European studies.

**Table 1:** Results from IAV screening and isolation of samples taken in Portugal 2008–2009.

Host species	Number of samples	AIV-positives	Prevalence	Subtypes Identified
		(RRT-PCR)	(%)	(Number of isolates)
Mallard (*Anas platyrhynchos*)	1542	69	4.5	H1N1 (2)
				H3N2 (1)
				H3N8 (2)
				H4N6 (2)
				H6N5 (1)
				H9N2 (2)^a^
				H10N7 (10)
				H11N3 (1)
Eurasian Teal (*Anas crecca*)	56	2	3.6	
Northern Shoveler (*Anas clypeata*)	30	0	0	
Eurasian Wigeon (*Anas penelope*)	12	0	0	
Gadwall (*Anas strepera*)	8	1	12.5	
Tufted Duck (*Aythya fuligula*)	5	0	0	
**Total**	**1653**	**72**	**4.4**	**8** b

a The sequences of two additional H9N2 viruses were determined by sequencing the viral H and N segments in the original sample.

b Number of different subtypes identified.

## Methods

### Ethics Statement

Ethical approvement for the study was obtained from the Linköping Animal Ethics Board (permit numbers 43-09 and 83-10). License to capture and ring birds was obtained from Instituto da Conservação da Natureza e da Biodiversidade (ICNB), Portugal (permit number 40/2008 and 40/2009).

**Figure 2:**
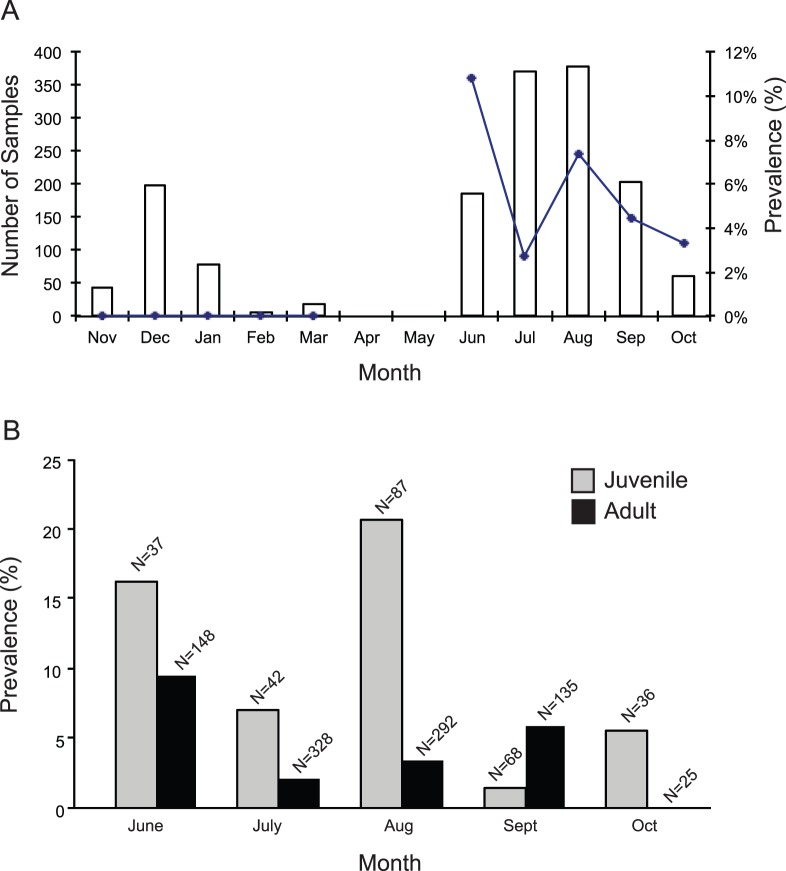
Temporal variation in IAV prevalence among Portuguese ducks during the period 2008–2009. (A) Overall temporal variation in IAV prevalence among sampled ducks. (B) The percentage of IAV-positives among juvenile and adult ducks in Portugal. Prevalence numbers are derived from sample screenings where IAV was detected by RRT-PCR.

### Duck Sampling

Ducks were captured and sampled between November 30^th^, 2008 and October 4^th^, 2009 in two Portuguese coastal wetlands located on the east Atlantic migratory flyway (Figure 1A), the São Jacinto Dunes Nature Reserve and the Taipal marshes (Figure 1B). Both locations represent refuge areas for ducks on the Vouga and the Mondego Lowlands, respectively [36]. The São Jacinto area includes a man-made pond, while Taipal is a 50 hectare fresh-water march that was previously used for cultivation of rice. Cloacal swab samples were placed in 1 ml tubes containing viral transport media composed of Hank’s balanced salt solution supplemented with 0.5% lactalbumin, 10% glycerol, 200 U/mL penicillin, 200 µg/mL streptomycin, 100 U/mL polymyxin B sulfate, 250 µg/mL gentamicin, and 50 U/mL nystatin; and stored at −80°C until analysed.

**Figure 3:**
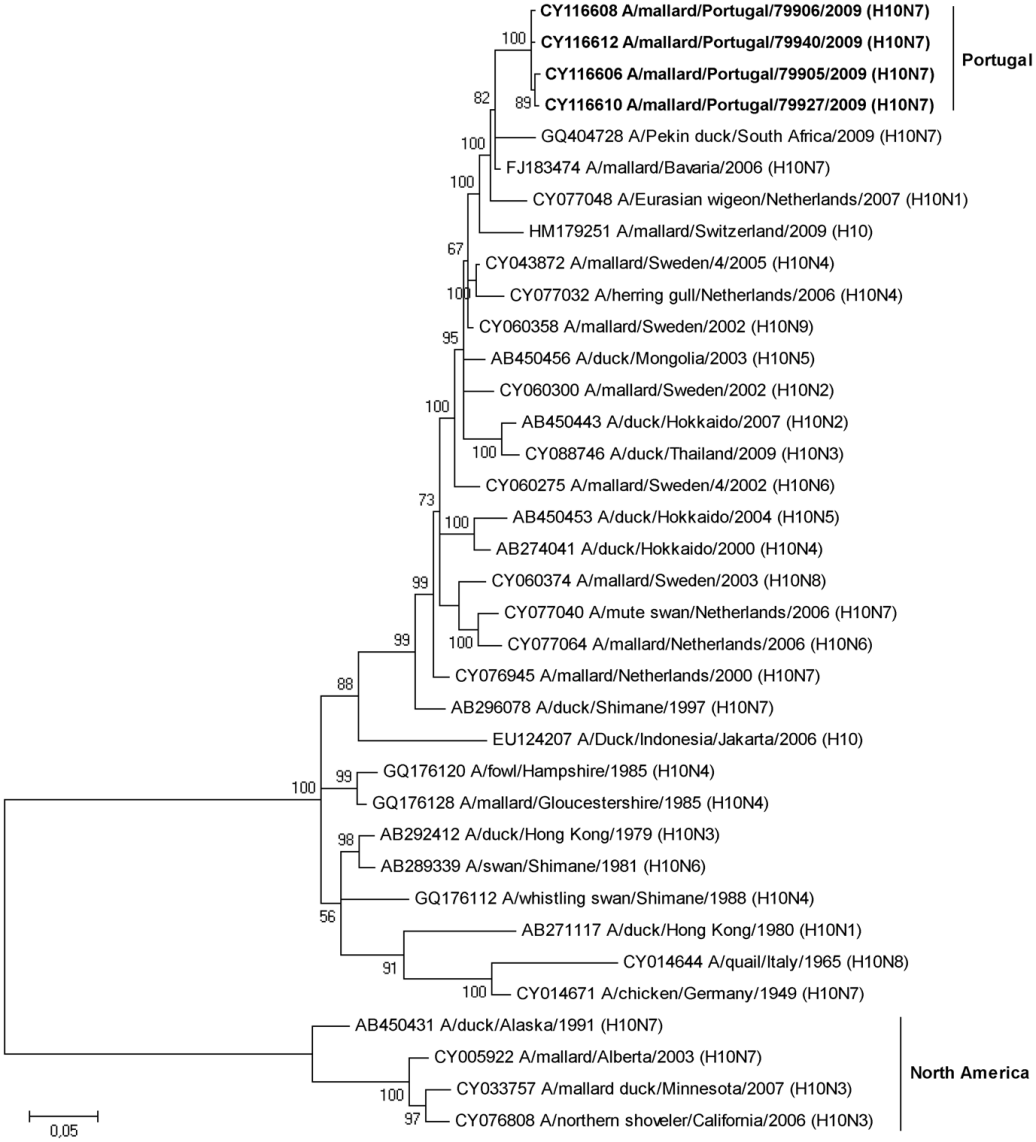
Bayesian phylogenetic tree showing relationships among H10 influenza A viruses isolated in this study and publicly available sequences from GenBank. The clades containing Portuguese and North American sequences are indicated, and Portuguese isolates are shown in bold. The tree is rooted at the midpoint and numbers at nodes indicate posterior probability support. The scale bar indicates substitutions per site.

**Figure 4:**
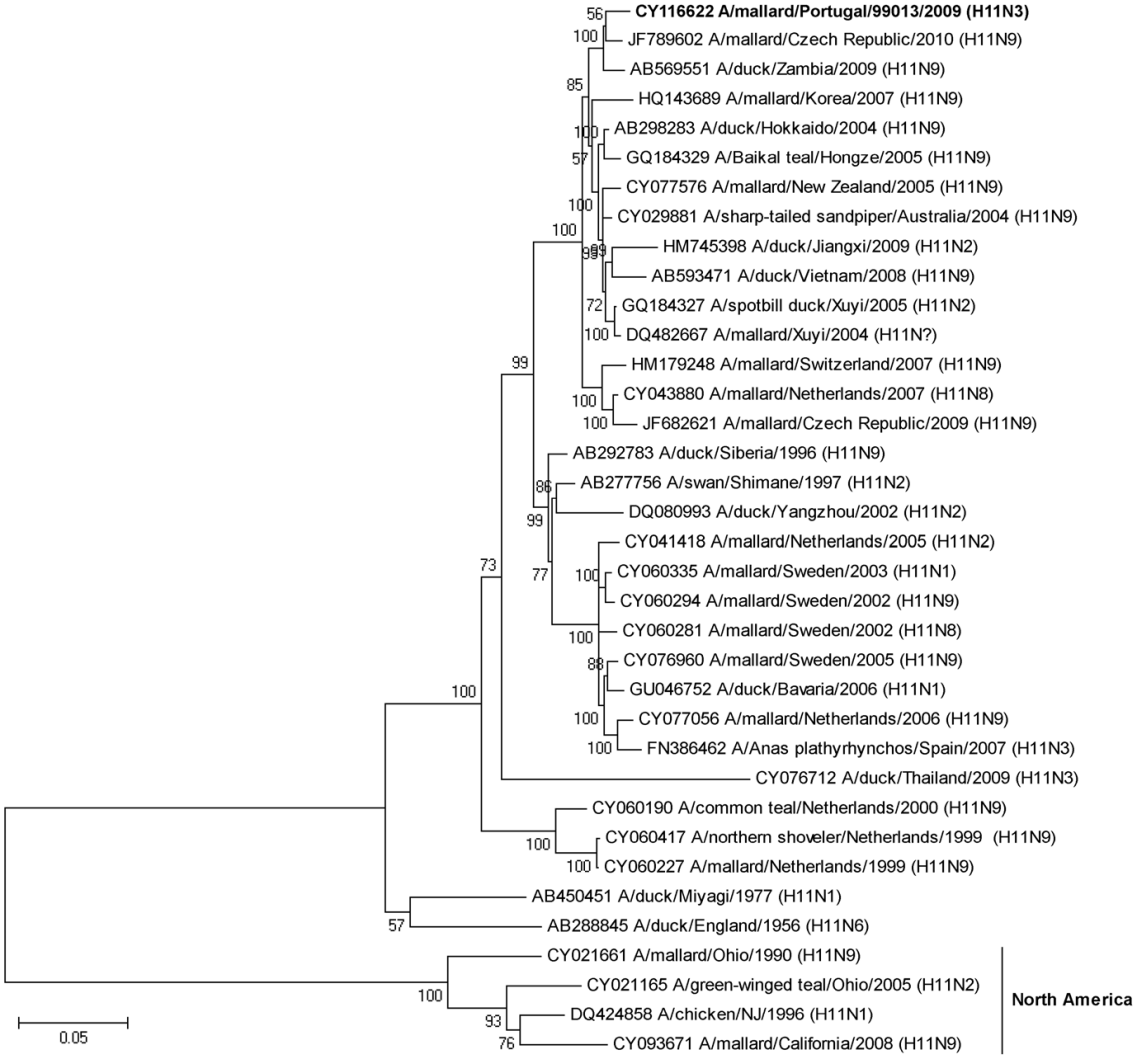
Bayesian phylogenetic tree showing relationships among H11 influenza A viruses isolated in this study and publicly available sequences from GenBank. The Portuguese sequence is shown with bold font and the clade containing North American sequences is indicated. The tree is rooted at the midpoint and numbers at nodes indicate posterior probability support. The scale bar indicates substitutions per site.

**Figure 5:**
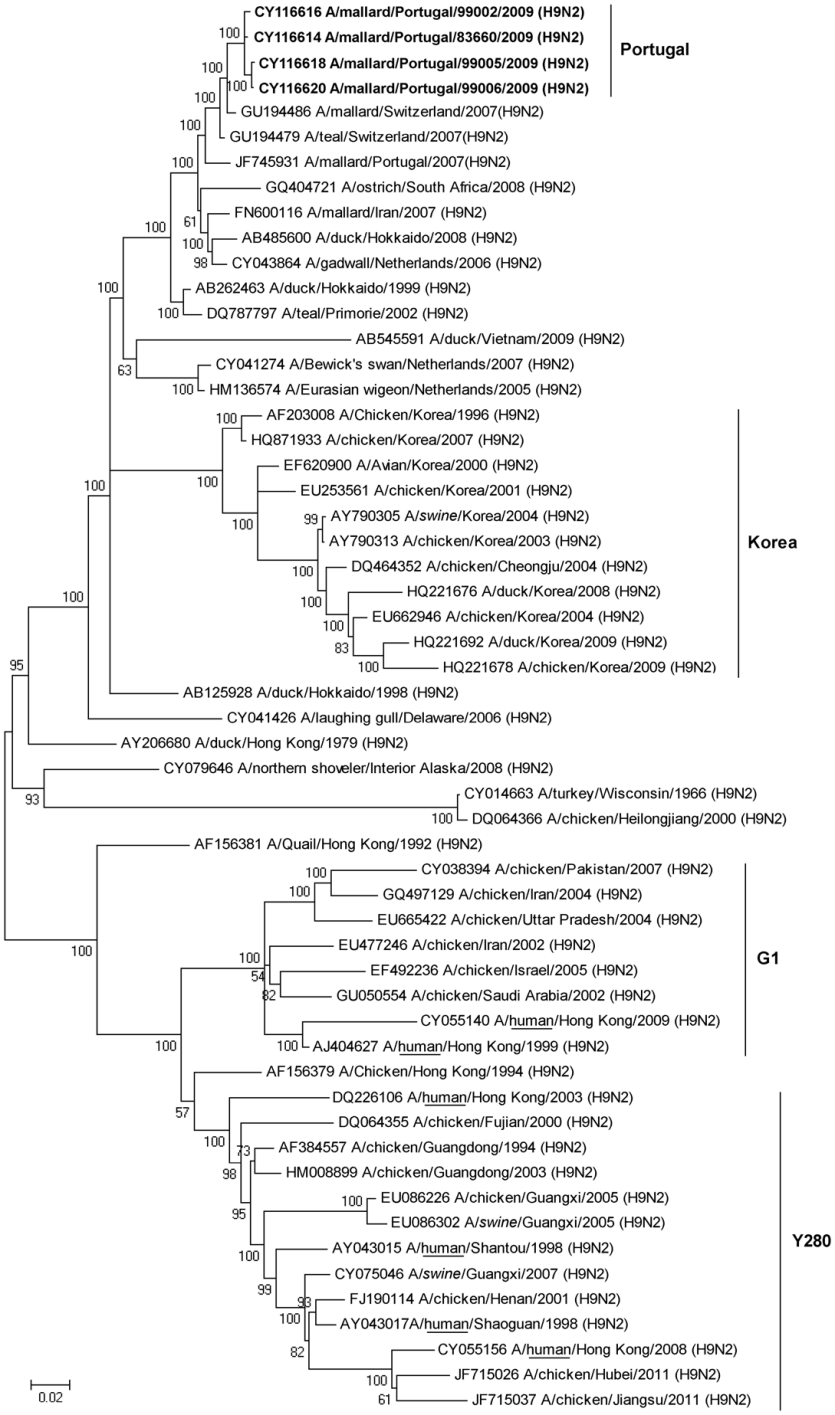
Bayesian phylogenetic tree showing relationships among H9 influenza A viruses isolated in this study and publicly available sequences from GenBank. The clade including the Portuguese sequences, as well as some well-established H9N2 linages (Korea, G1 and Y280) are indicated. In addition, isolates associated with infections in humans and swine have been indicated by underlining and italics, respectively. The tree is rooted at the midpoint and numbers at nodes indicate posterior probability support. The scale bar indicates substitutions per site.

### Virus Detection, Isolation and Subtyping

IAV was screened for using a standard TaqMan-based real-time reverse transcriptase PCR (RRT-PCR) method [37]. RNA was extracted from 100–200 µl of the sample material using automatic extraction instruments: either the NucliSens® easyMagTM (bioMérieux, Norge AS, Oslo, Norway) or, the Qiagen M48 robot in combination with the Viral Mini Extraction kit (Qiagen, Germantown, MD, USA). Prior to egg culturing, IAV-positive samples were screened for H5 and H7 subtype virus according to previously established protocols [37], in order to ensure that no high-pathogenic virus was propagated. IAV positive samples were inoculated into 11-day-old embryonated, specific pathogen free (SPF), chicken eggs to propagate virus. The subtypes of the cultured IAV isolates were determined by Hemagglutination Inhibition assay using antisera against the different HA variants as described previously [38], or by sequencing amplified HA and NA segments using established methods [39], [40], [41] either directly or after being cloned into a pGEM-T vector (Promega, Madison).

### Phylogenetic Analysis

Assembly and editing of consensus nucleotide sequences of IAVs were performed using Geneious v5.5 [42]. Sequences generated in this study were deposited in GenBank under the accession numbers: CY116606-CY116623. HA and NA sequences of virus from the Portuguese ducks were aligned with sequences from public databases using the MAFFT algorithm [43]. Sequences from databases were selected based on similarity to sequences of this study as indicated by BLAST, or for the more distantly related taxa, as representatives of distinct clades observed in initial large trees constructed with large numbers of IAV sequences. The appropriate nucleotide substitution model for phylogenetic analyses (i.e., the GTR G+I model for the H10 tree, the HKY G+I model for the H10 tree and the GTR G model for the H11 tree) were determined using the jModelTest v 0.1 software [44]. Phylogenetic relationships between IAV nucleotide sequences were inferred with MrBayes v 3.1.2 [45], and by using a sufficient number of generations and sample frequencies to obtain standard deviation of split frequencies below 0.01. For the consensus trees, Bayesian posterior probabilities were calculated after excluding 25% of sample trees as burn-in. The H9, H10 and H11 tree was inferred based on a 1488 bp (52–1539), a 942 bp (721–1662) and a 1658 base pair fragment of the HA segment, while the N2, N3 and N7 trees were based on 1320 bp (46–1365), 1120 bp (199–1318) and 866 bp (424–1289) base pair fragments, respectively.

## Results

### IAV Prevalence in Birds

Screening of 1653 birds in two Portuguese wetlands during a 1-year period from 2008 to 2009 resulted in 72 RRT-PCR positive samples, corresponding to a prevalence of 4.4%. These positive samples were taken from 68 different individuals, representing six different waterfowl species. Among IAV-positive birds, all except two samples taken from Eurasian Teals and one sample from a Gadwall (*Anas strepera*), were collected from Mallards (Table 1). During the first half of the sampling period (November 2008 to March 2009), a total of 435 birds were sampled, however, when screen by RRT-PCR, none of these birds tested positive for IAV (Figure 2a). The first viruses were detected in June 2009, during what seems to have been a prevalence peak, with 10.8% of sampled birds infected (Figure 2a). Later in the autumn 2009 the IAV prevalence declined. Overall, among infected birds across the surveillance period, first year ducks had a higher virus prevalence compared with adult birds (Figure 2b). Some birds had complex infection patterns, for example, a female Mallard, which was sampled four times between 9 September and 22 September and tested negative, positive, negative and positive again for IAV.

### Subtype Diversity

Eight different subtypes were detected across 21 isolates, including H1N1, H3N2, H3N8, H4N6, H6N5, H9N2, H10N7, and H11N3, and among these were H10N7 and H9N2 the most predominant (Table 1). Additionally, a number of viruses have been sequenced directly from the original sample and for which only the HA- or-NA-subtype has been determined, including H1, H3, H4 and N2 (data not shown). However, as determined by subtype specific RRT-PCR, no H5 or H7 viruses were identified.

### Phylogenetic Analysis

In total, the complete or partial sequences of the HA and the NA gene segment were determined for nine isolates with subtypes H10N7, H9N2 and H11N3. These subtypes were selected for sequencing because they either represented a large fraction of the total number of isolates (i.e., the H10N7 isolates with sample identities 79905, 79940, 79927 and 79940), they were potentially interesting from a transatlantic perspective (the H11N3 isolate, from sample 99013), or constitutes a subtype with emerging pandemic potential (H9N2 from samples 83660, 99002, 99005 and 99006). All H10N7 viruses came from samples collected on June 7, 2009, while the H9N2 virus samples were taken during a period from June 17 to August 8, 2009.

Phylogenetic relationships between sequences generated in this study and sequences in GenBank were analysed (Figures 3, 4, 5, S1, S2, S3). Broadly, in the H10 tree (including isolates 79905, 79906, 79927 and 79940) and the H11 tree (with Portuguese isolate, 99013) there is a distinct division between Eurasian and American virus lineages (Figures 3 and 4). However, within the Eurasian clade of these trees, H10 and H11 sequences from Portugal clustered with sequences from African viruses as well as with viruses isolated in Central- and Northern Europe. The HA sequence of the H10N7 virus showed 97.2% sequence identity with HA of a H10N7 virus isolated from a Mallard in Bavaria 2009, and 95.6% similarity with a South African duck virus from 2003. Similarly, the HA sequence of the Portuguese H11N3 virus had a pair-wise identity of 98.2% and 97.9% with a H11N9 virus isolated form a Mallard in the Czech Republic in 2010 and with viruses from a duck and a goose in Zambia in 2009. Initial sequence identity analyses of the HA sequences of the four H9N2 viruses revealed some similarity to H9N2 viruses isolated from swine, and sequences of virus isolated from swine are included in the H9 tree as well as in the N2 tree (Figures 5 and S2). However, the H9 tree illustrates that the Portuguese sequences were most closely related to sequences of viruses from Switzerland, Portugal, South Africa, Iran, Eastern Russia and Japan, isolated between 1999 and 2008 from anatids and from a farmed South African Ostrich. Moreover, the H9 tree showed that viral sequences from the Portuguese birds did not belong to any previously established H9N2 linage (Figure 5).

Phylogenetic trees of the NA sequences illustrated similar patterns of interhemispheric similarities. The NA sequence of the Portuguese H10N7 isolates clustered with sequences of European H7N7 viruses from wild waterfowl isolated in Slovenia and Hungary and the NA sequence of a South African H10N7 virus from a farmed Peking duck (Figure S1). The N2 sequence tree illustrates that the H9N2 Portuguese virus sequence clustered with Asian H9N2 virus from duck and chicken as well as with a virus isolated from a South African Ostrich (Figure S2). Finally, N3 sequences of the H11N3 virus clustered with sequences of Asian H11N3 and H5N3 viruses from wild ducks (Figure S3).

## Discussion

Despite intense surveillance efforts to map the epidemiology of IAV, our understanding of the global distribution of this virus as well as the circulation of its different subtypes is still limited. To better understand how IAV is transmitted and spread, it is important to monitor virus occurrence in wild birds for prolonged periods of time at different geographic locations. Moreover, mapping of global IAV distribution in geographic areas which are stopover sites along bird migratory flyways is of particular interest [46]. In this study, Portuguese waterfowl were screened for IAV during a one-year period from 2008–2009. Samples were taken from different species of waterfowl which, although they show variation in migratory behaviour, including ‘choice’ of migratory pathways, share basic traits in terms of seasonal migration (Table 1). Large populations of Mallard, Eurasian Wigeon, and Eurasian Teal that breed in the northern parts of Europe and Asia, including Scandinavia and eastward through Siberia, winter in southern Europe and northern Africa, where they mix with resident conspecifics. Similarly, Tufted Ducks from central Europe through Siberia will, during winter season, mix with duck populations resident in the Iberian Peninsula. The possibility of contact between migratory birds and Portuguese resident birds also applies to the Gadwall and Northern Shoveler sampled in this study, despite the fact that these birds have a more patchy distribution throughout Europe [47]. The sampling locations, São Jacinto and Taipal, are parts of wide branching river systems that are important breeding sites for waterfowl, as well as stopover sites along the European-African migratory flyway [1], making them meeting locations for populations of stationary, migrating and wintering birds.

The surveillance results, including an overall IAV prevalence of 4.4% among sampled waterfowl, of which the absolute majority were Mallards, corresponds with results from previous studies in the region [32], but is significantly lower than prevalence numbers seen in studies conducted further north in Europe [22]. In both Northern Europe and North America there is a characteristic prevalence peak of LPAI in ducks in the autumn [2], [22], [25], [26], [30], [48], [49], likely due to large densities of immunological naïve birds during early stages of migration [2]. The high IAV prevalence in Mallard pulli and young juveniles observed in June in our study is noteworthy, possibly indicating that IAV do not primarily arrive with migrating birds from the northern parts of Europe, but instead circulate in breeding populations in Mediterranean wetlands. Previous avian influenza screenings on the Iberian Peninsula, in Portugal and Spain, between the years 2005–2009, have shown that overall 5 – 10% of Mallards and 15% of Eurasian Teals were IAV positive [32], [50]. In Portugal, the total prevalence among a number of different bird species tested was 1.6%, hence slightly lower than the 4.4% prevalence presented in this study. These differences in prevalence might be related to natural temporal variation at different sampling locations, but is possibly also related to the fact that Henriquens and colleagues [50] sampled thirteen different orders of birds, some of which regarded less susceptible to IAV, hence bringing the overall number of infected birds down, whereas our study was rather focused on samples from waterfowl which is a well-established and important IAV reservoir. Several European studies have shown that the IAV prevalence in birds is high throughout the autumn, starting in August, with peaks in October – November [22]. In addition, the IAV prevalence presented in this study reinforces observations of a lower IAV prevalence in birds on more southern latitudes (*i.e.*, locations with warmer climates) compared to the prevalence in birds in northern Europe [22], [32]. However, when interpreting surveillance data, it is important to note, that the virus prevalence might fluctuate significantly during seasons between different years. Studies in Europe [3], Japan [51] and North America [52], have shown that the IAV prevalence drops significantly during the winter, which is also reflected in the absence of positive samples among the few samples collected from November – March. The low number of ducks sampled during this period was partly due to the floods that occurred in the region.

The most common subtypes detected from waterfowl in Taipal and São Jacinto in 2008 and 2009 were the H10N7 and H9N2 combinations. Previous studies in Europe have shown that H4, H6 and H7 are the most common HA subtypes in wild Mallards, with frequencies ranging from 10% for H6 and H7 to 16% for H4 [3]. In contrast, H9 and H13–16 are rare in waterfowl, including Mallards. Correspondingly, among the NA subtypes most often isolated from Mallard, N2 and N6–N9 are found in 10–20% of IAV-infected birds. Moreover, in the north-eastern parts of Spain, H4N6 has reappeared in annual surveys [32]. In Portugal, during 2005–2009, the H4N6 together with H1N1 were the predominant subtypes, corresponding to approximately 15% of all viruses that were isolated [50].

Portugal is situated in the east Atlantic migratory flyway, an important migratory route that connects Eurasian and African bird populations [1]; indeed this geographic location is reflected in phylogenetic trees inferred in this study. Viral sequences from the Portuguese birds show high similarity with viruses from Africa, Europe and Asia, reflecting the regions geographic location, positioned along a major migratory flyway between Eurasia and Africa. The HA and NA sequences from the Portuguese birds provide evidence of transmission of viruses between Europe and Africa, but not between Eurasia and North America, although such intercontinental viral gene flow has been observed previously [33]. The importance of screening and sequencing of IAVs are shown by the fact that sequences of viruses isolated in Portugal show high similarity to sequences involved in pathogenic outbreaks elsewhere on the globe. First, both the HA and NA segments of the H10N7 virus were similar to a South African H10N7 virus, isolated from a Peking duck on a farm in 2009, during one of the most recent outbreaks of avian influenza in this country [53]. Second, the H11N3 HA segment of the Portuguese isolate clustered with two H11N9 viruses isolated in Zambia in 2009 from wild waterfowl [54]. This study describes close genetic relationships between viruses isolated from wild birds in Zambia and virus isolated from South African poultry. It is noticeable that the African sequences, although they constitute only one-fifth or one-ninth of the number of European H10 and H11sequences available in the NCBI database, show up in the H10 and H11 tree, and that they seem to cluster with sequences from Portugal. Possibly, this lack of IAV sequence information from African viruses has made it difficult, in previous studies in the region, to observe close phylogenetic relationships between European, in this instance Portuguese, and African viruses. The third hemagglutinin type analysed in this study, the H9 (of the H9N2 subtype) have reached a near panzootic distribution in Eurasia, as well as being established in domestic poultry in Germany, Italy, Pakistan, Saudi Arabia, South Africa and the USA [19], [20]. Other studies have shown that H5N1 and H9N2 virus co-circulate among farmed birds at food markets in China, and that many of the H9 subtype viruses found in chicken seemed to be products of reassortment events involving H5N1-related viruses [55]. In addition, several cases of human H9N2 infections have been reported [56], [57]. The potential to break host species barriers can be observed in the H9 tree inferred in this study, in which virus sequences from swine along with sequences associated with human H9N2 infections cluster with viral sequences from chicken. However, it is important to note that the IAVs associated with avian influenza infections in humans are found within one major linage of the tree, together with other Asian viruses, while the Portuguese H9N2 virus are found within the other major branch of the tree, formed by Eurasian and African viruses.

Surveillance and characterization of viruses from regions that are important migratory stopover sites are necessary in order to increase our understanding of how of IAV gene segments are spread across the globe. Stopover sites that are also crossroads for migratory birds from different regions are particularly important, as the interaction of large populations of birds from different geographic locations may have implications for the emergence and spread of new IAVs. This study highlights the importance of Portugal as a migratory crossroad for IAV gene segments, and accordingly, a possible route by which viruses are spread between Europe and Africa, as well as a location where new virus variants might emerge. In addition, this study reinforced previous observations that the IAV prevalence among Mallards, in southern Europe, is highest during the summer month, something that needs to be considered in future surveillance programs.

## Supporting Information

Figure S1 **Bayesian tree based of the N7 subtype nucleotide sequences.** The N7 sequences from Portuguese H10N7 viruses isolated in this study are indicated in bold. In the phylogenetic analysis, the HKY G+I model of nucleotide substitution was used. The tree is rooted at the midpoint and numbers at nodes indicate the posterior probability node support. Scale bar indicates substitutions per site.
(TIF)

Figure S2 **Bayesian tree of the N2 nucleotide sequences.** The N2 sequences from Portuguese H9N2 isolates are indicated in bold. In the phylogenetic analysis, the GTR G model of nucleotide substitution was used. Numbers at nodes in the midpoint rooted tree indicate the posterior probability node support. Scale bar indicates substitutions per site.
(TIF)

Figure S3 **Bayesian tree of the N3 subtype nucleotide sequences.** The N3 sequence from the Portuguese H11N3 isolate is indicated in bold. For the phylogenetic analysis, the GTR G model of nucleotide substitution was used. Numbers at nodes in the midpoint rooted tree indicate the posterior probability node support. Scale bar indicates substitutions per site.
(TIF)
