# Supplementary figures and images for: Birds and Viruses at a Crossroad - Surveillance of Influenza A Virus in Portuguese Waterfowl
Source: PLoS One. 2012 Nov 7;7(11):e49002. doi: 10.1371/journal.pone.0049002 (PMC3492218; doi:10.1371/journal.pone.0049002)

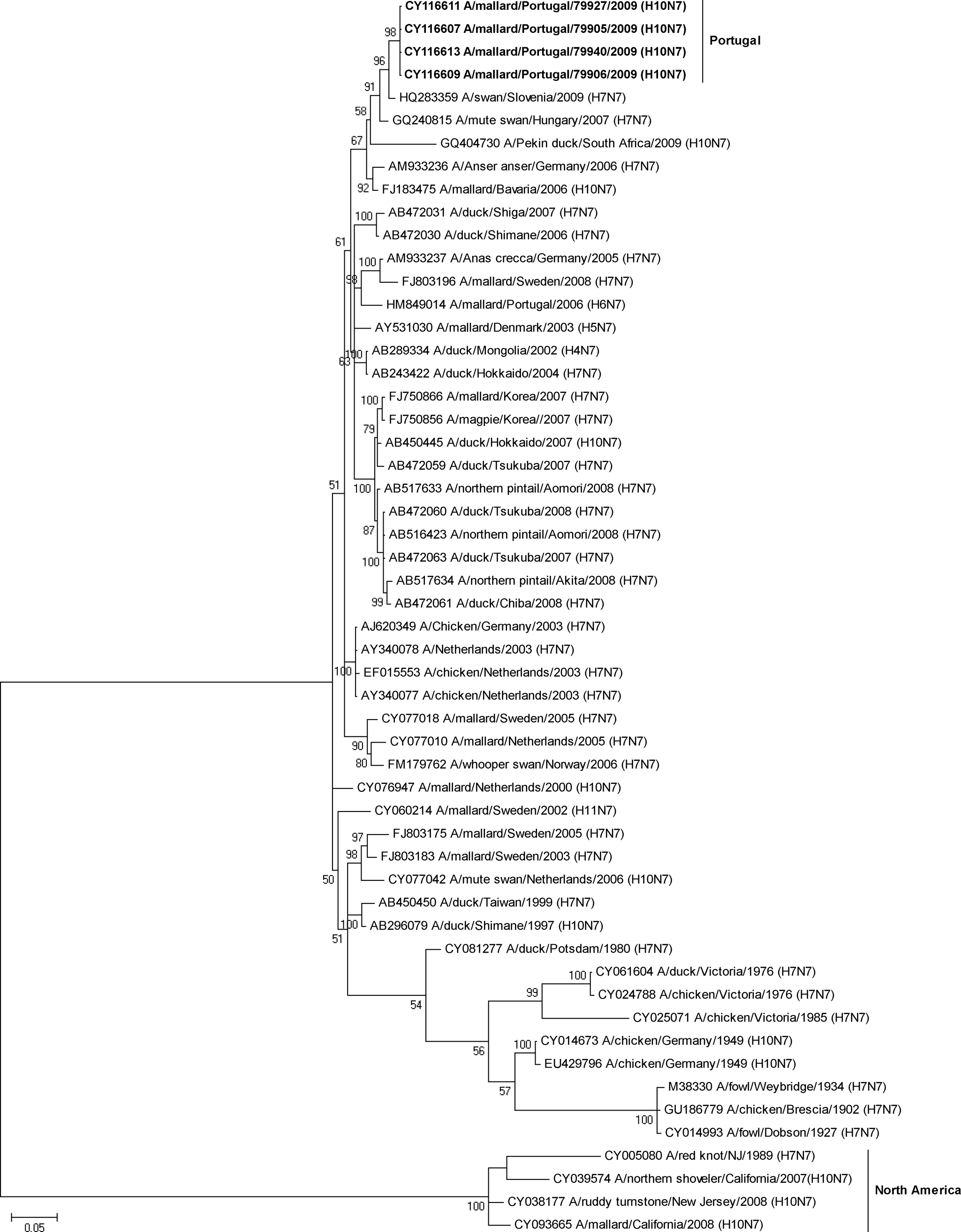

Supplement: Figure S1 — Bayesian tree based of the N7 subtype nucleotide sequences. The N7 sequences from Portuguese H10N7 viruses isolated in this study are indicated in bold. In the phylogenetic analysis, the HKY G+I model of nucleotide substitution was used. The tree is rooted at the midpoint and numbers at nodes indicate the posterior probability node support. Scale bar indicates substitutions per site. (TIF) [file pone.0049002.s001.tif]

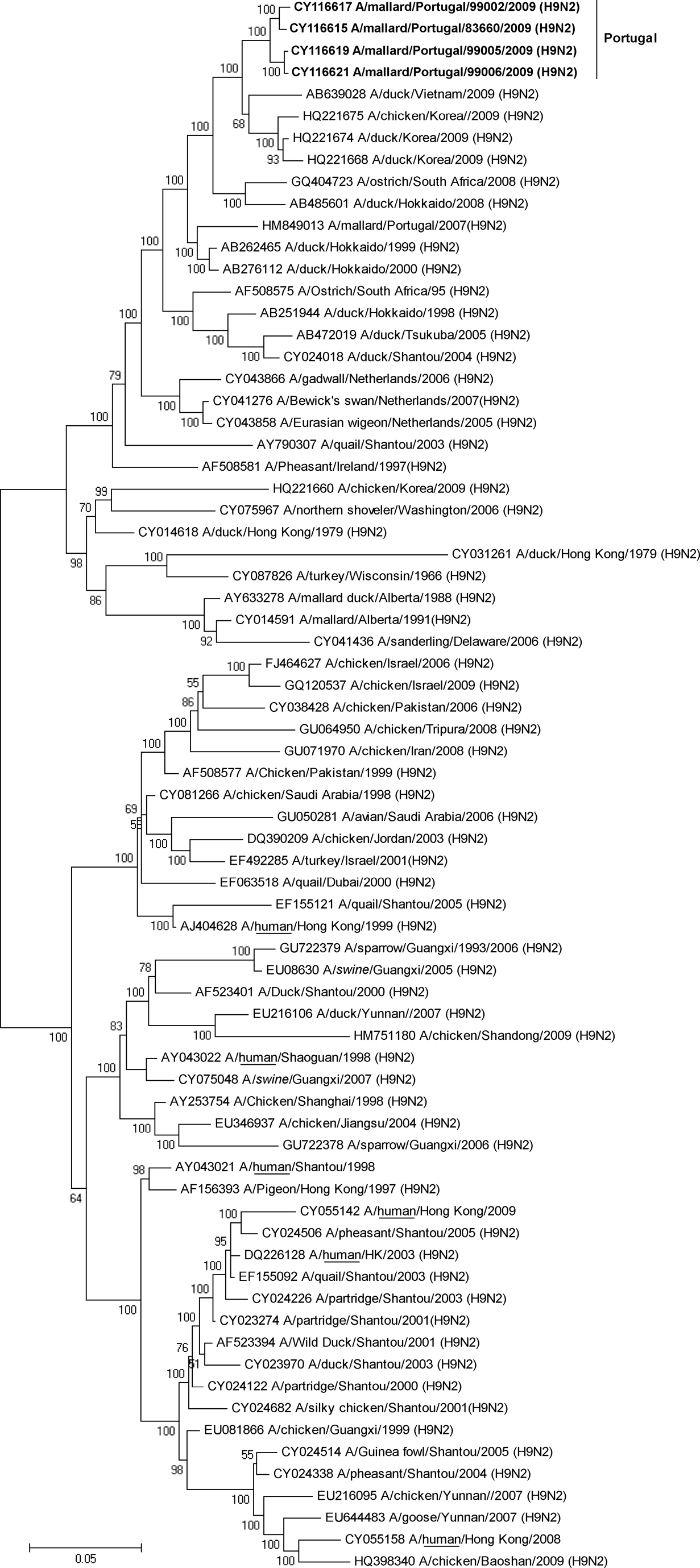

Supplement: Figure S2 — Bayesian tree of the N2 nucleotide sequences. The N2 sequences from Portuguese H9N2 isolates are indicated in bold. In the phylogenetic analysis, the GTR G model of nucleotide substitution was used. Numbers at nodes in the midpoint rooted tree indicate the posterior probability node support. Scale bar indicates substitutions per site. (TIF) [file pone.0049002.s002.tif]

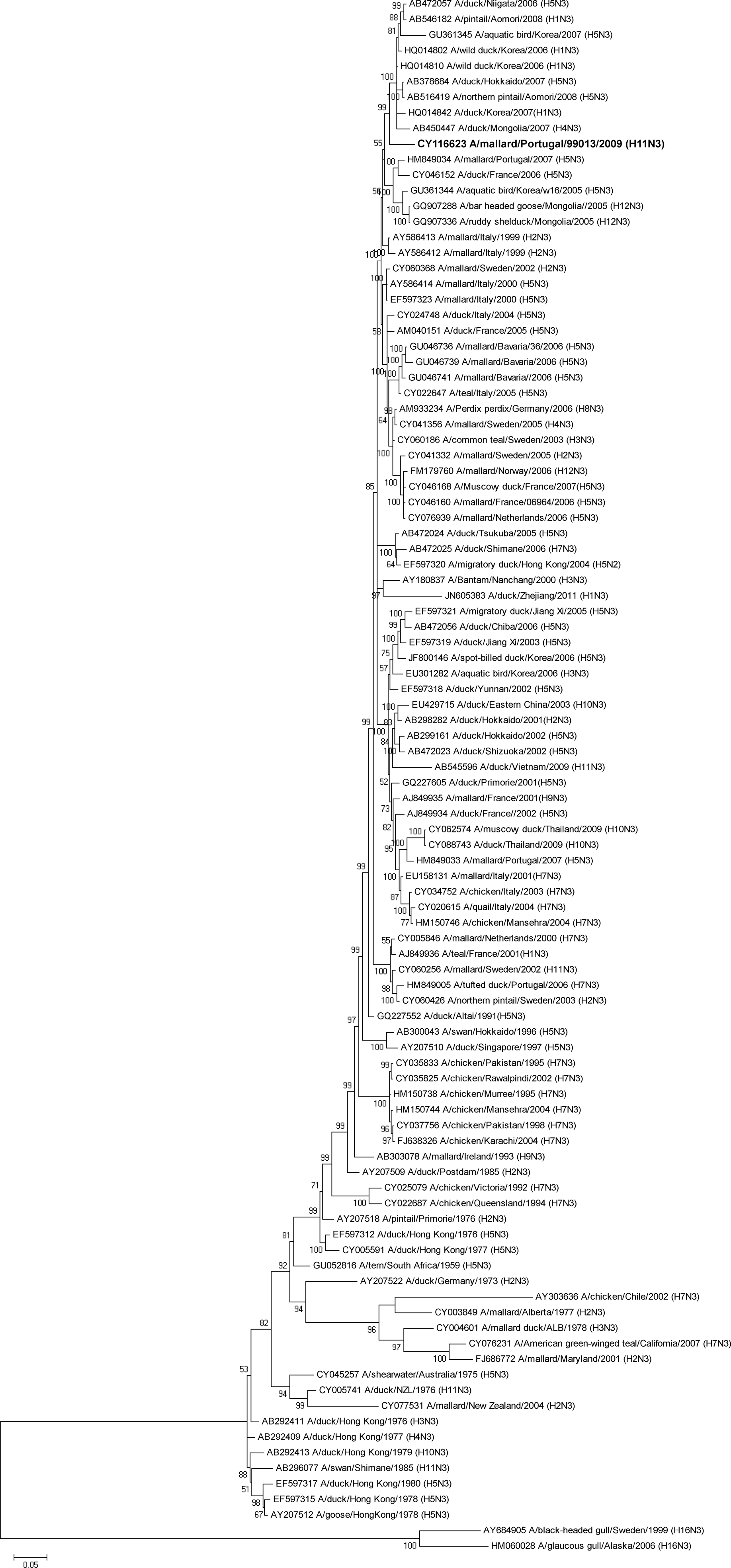

Supplement: Figure S3 — Bayesian tree of the N3 subtype nucleotide sequences. The N3 sequence from the Portuguese H11N3 isolate is indicated in bold. For the phylogenetic analysis, the GTR G model of nucleotide substitution was used. Numbers at nodes in the midpoint rooted tree indicate the posterior probability node support. Scale bar indicates substitutions per site. (TIF) [file pone.0049002.s003.tif]
